# Mathematical modeling study of school-based chlamydia screening: potential impact on chlamydia prevalence in intervention schools and surrounding communities

**DOI:** 10.1186/s12889-020-09466-y

**Published:** 2020-09-05

**Authors:** Minttu M. Rönn, Richard Dunville, Li Yan Wang, Meghan Bellerose, Yelena Malyuta, Nicolas A. Menzies, Maria Aslam, Felicia Lewis, Cherie Walker-Baban, Lenore Asbel, Sarah Parchem, Lisa Masinter, Ernestina Perez, Tom L. Gift, Katherine Hsu, Lisa C. Barrios, Joshua A. Salomon

**Affiliations:** 1 Department of Global Health and Population, Harvard T. H. Chan School of Public Health, Boston, USA; 2 Division of Adolescent and School Health, National Center for HIV/AIDS, Viral Hepatitis, STD, and TB Prevention, Centers for Disease Control and Prevention, Atlanta, GA USA; 3 Office of the Director, National Center for HIV/AIDS, Viral Hepatitis, STD, and TB Prevention, Centers for Disease Control and PreventionPrevention, Atlanta, USA; 4 Division of STD Prevention, National Center for HIV/AIDS, Viral Hepatitis, STD, and TB Prevention, Centers for Disease Control and Prevention, Atlanta, USA; 5 STD Control Program, Philadelphia Department of Public Health, Philadelphia, PA USA; 6 Bureau of Maternal, Infant, Child, and Adolescent Health, Chicago Department of Public Health, Chicago, USA; 7 Office of Student Health and Wellness, Chicago Public Schools, Chicago, USA; 8 Division of STD Prevention & HIV/AIDS Surveillance, Massachusetts Department of Public Health, Jamaica Plain, USA; 9 Center for Health Policy / Center for Primary Care and Outcomes Research, Stanford University, Stanford, USA

**Keywords:** Chlamydia, Screening, Adolescent health

## Abstract

**Background:**

Chlamydia screening in high schools offers a way to reach adolescents outside of a traditional clinic setting. Using transmission dynamic modeling, we examined the potential impact of high-school-based chlamydia screening programs on the burden of infection within intervention schools and surrounding communities, under varying epidemiological and programmatic conditions.

**Methods:**

A chlamydia transmission model was calibrated to epidemiological data from three different settings. Philadelphia and Chicago are two high-burden cities with existing school-based screening programs. Rural Iowa does not have an existing program but represents a low-burden setting. We modeled the effects of the two existing programs to analyze the potential influence of program coverage and student participation. All three settings were used to examine a broader set of hypothetical programs with varying coverage levels and time trends in participation.

**Results:**

In the modeled Philadelphia program, prevalence among the intervention schools’ sexually active 15–18 years old population was 4.34% (95% credible interval 3.75–4.71%)after 12 program years compared to 5.03% (4.39–5.43%) in absence of the program. In the modeled Chicago program, prevalence was estimated as 5.97% (2.60–7.88%) after 4 program years compared to 7.00% (3.08–9.29%) without the program. In the broader hypothetical scenarios including both high-burden and low-burden settings, impact of school-based screening programs was greater in absolute terms in the higher-prevalence settings, and benefits in the community were approximately proportional to population coverage of intervention schools. Most benefits were garnered if the student participation did not decline over time.

**Conclusions:**

Sustained high student participation in school-based screening programs and broad coverage of schools within a target community are likely needed to maximize program benefits in terms of reduced burden of chlamydia in the adolescent population.

## Background

Sexually transmitted infections (STIs) are prevalent among adolescents and young adults [1, 2]. However, a number of factors may contribute to relatively low STI screening rates among adolescents, including relatively low use of preventive care generally [3], as well as concerns about confidentiality that may inhibit use of sexual and reproductive health services more specifically [4]. There are a number of school-based STI screening interventions, which have been or are currently implemented in high schools across the United States [5–14]. A review of STD screening programs in high schools called for increased effort to understand community factors [8], and noted that it has been difficult to determine the impact of school-based screening on chlamydia transmission dynamics for the school population and for the community.

Typically, screening events are implemented in collaboration with local public health departments, or screening is offered at school-based health centers, with the aim in either case to screen the entire school population within a short amount of time (e.g. 1 week) [12]. School-based chlamydia screening may improve case-finding and treatment among infected individuals [15]. A recent evaluation of the screening events in Detroit schools found that the events were associated with a reduction in chlamydia positivity [16]. A previous dynamic transmission model, analyzing 4 years of data from a screening program in Philadelphia high schools, suggested that screening both males and females could reduce chlamydia prevalence within participating schools [10]. The study focused on outcomes within the intervention schools and did not examine the long-term impact of screening programs on community-level chlamydia transmission dynamics.

In this study, we used mathematical modeling to evaluate the potential impact of school-based screening programs on chlamydia transmission dynamics, accounting for various epidemiological factors and programmatic features that may influence the program effects both within schools and in the surrounding communities. By modeling an array of different program scenarios in both high-burden and low-burden settings, we examined how specific features from existing programs, as well as possible implementation scenarios for hypothetical programs, could affect chlamydia transmission dynamics in different epidemiological contexts.

## Methods

### Mathematical model framework

**Fig. 1:**
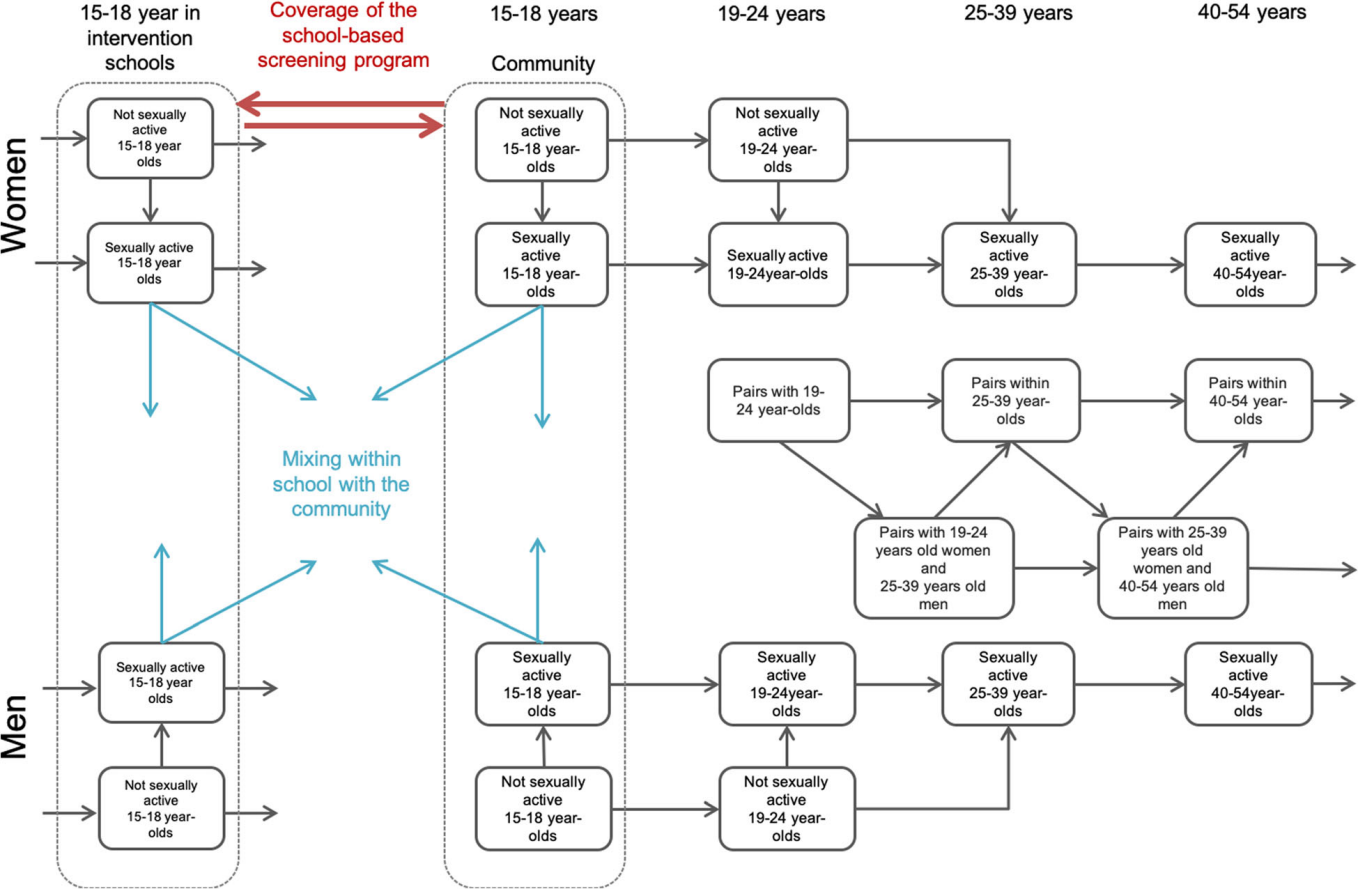
Model demographics, sexual behavior and partnership formation

The model in this study expanded on a previously developed chlamydia transmission model, described in detail elsewhere [17]. A deterministic, compartmental pair-formation modeling approach was used, in which the population living in a defined location was divided into a range of different categories, reflecting stratification by age (15-18y, 19-24y, 25-39y, 40-54y), by sex (heterosexual men and women), by sexual partnership category (never had sex, sexually active single people, and people in a long-term partnerships), and by sexual activity level (higher and lower sexual activity defined by frequency of short-term relationships). Long-term partnerships were represented as distinct compartments in the model, stratified based on the age and infection status of both partners. For this study, we expanded the modeling framework by dividing the 15–18 year-old population into those in intervention schools and those outside of the intervention schools but in the surrounding communities (Fig. 1). In the two youngest age groups (15-18y and 19-24y), the model included a fraction of the population that was not yet sexually active, with age-specific rates of transition into the sexually active population; these rates were time-varying in the youngest age group to allow for changes in the population at-risk for chlamydia. We assumed that all people ages 25 and older were sexually active. Sexually active single people of any age in the model could have short-term relationships; sexually active people older than 18 years could enter long-term partnerships. Chlamydia infection status was represented using a susceptible-infected-susceptible structure.

### Settings

We applied the model in three different settings, spanning a range of reported chlamydia diagnosis rates. Philadelphia and Chicago are large cities with relatively high rates of chlamydia diagnoses and with existing high-school-based screening programs. Rural Iowa has relatively low chlamydia diagnosis rates and does not have an existing school-based screening program. Among women aged 15-24y, rates of reported chlamydia diagnoses in 2015 were 7433 per 100,000 in Philadelphia [18], 6715 per 100,000 in Chicago [19] and 2807 per 100,000 in the state of Iowa [20], compared to 3376 per 100,000 nationally [21]. For our analysis, we calculated reported diagnosis rates for Iowa excluding the 10 most populous counties to approximate the burden in more rural areas, which yielded a diagnosis rate of 1948 per 100,000 in 2015.

Philadelphia has the largest and longest-running high-school-based chlamydia screening program in the United States, established during the 2002–2003 school year. Between 2002 and 2015, there were 10,000–20,000 tests conducted annually, and the schools enrolled in the program covered approximately 55% of the 15–18-year-old population in the city. In Philadelphia’s intervention schools student participation declined over time, from approximately 30% of the student population during the 2002–2003 school year to 16% during the 2014–2015 school year. Chicago began a screening event program in 2010, following a pilot study in 2008. Between 2010 and 2013, the program was implemented in schools covering approximately 12.5% of the 15–18 year-old population in the city, and the number of tests conducted yearly increased from 2200 to 6900.

### Data and model calibration

Model calibration was undertaken using a Bayesian framework [22] operationalized with Incremental Mixture Importance Sampling [23]. Each setting was calibrated independently. The calibration approach yielded a joint posterior probability distribution for the parameter values, informed by a combination of specified prior distributions and the data likelihood. For a subset of parameters, we resampled from distributions defined by posterior estimates in our previously calibrated national model [17]. Table S1 in Supplement 1 describes each parameter that was varied in the calibration and its prior and posterior ranges. To estimate community-level chlamydia transmission dynamics in the absence of school-based screening programs, we calibrated the model to three sources of setting-specific data: reported chlamydia diagnosis rates, chlamydia positivity estimates among 15–18 years olds, and the proportion of the high school population who report having ever had sex.

Sex- and age-stratified chlamydia reported diagnosis rates were obtained for the three study settings [18–20], and the model was calibrated to data prior to the initiation of school-based screening programs in the cases of Philadelphia and Chicago. For Philadelphia, we used the average of diagnosis rates during 2002–2015 as a proxy measure, as there were no data available prior to 2002. For Chicago, we used diagnosis rates during 2000–2009. For Iowa (excluding the 10 most populous counties), we used the diagnosis rates during 2000–2015.

Both Philadelphia and Chicago provided estimates of chlamydia positivity in intervention schools. For calibration, we used positivity estimates from the first year available for each program as a proxy for the baseline chlamydia prevalence among the population of sexually active 15–18 year-olds in each city. For Iowa, we used a chlamydia positivity estimate from rural family planning clinics [24]. To calibrate rates of sexual initiation among 15–18 year olds, we used city-level data from the Youth Risk Behavior Survey (YRBS) [25] on the percentage of high school students who reported ever having had sex (state-level data used for Iowa).

The model was initially run to equilibrium using time-invariant parameters. We introduced time-varying parameters governing rates of sexual initiation starting in 1991, to correspond to years of data availability from YRBS, and time-varying parameters relating to screening rates and completeness of reporting on diagnosed infections in the general population starting in 2000, to correspond to the first year of case report data included in the likelihood.

### Analysis

We conducted two sets of analyses. In the first set of analyses, using only the models for Philadelphia and Chicago, we examined the potential influence of different observed programmatic features from the school-based screening programs in those cities on the estimated benefits from the programs. We used the calibrated models to estimate chlamydia prevalence in the 15–18 year-old population in the absence of school-based screening. We then estimated the potential impact of school-based screening using program data on the fraction of the 15–18 year-old population enrolled in intervention schools (“coverage”) and on student participation within intervention schools over time as input parameters. We used information on the highest and lowest yearly participation levels observed in Chicago and Philadelphia to determine ranges of participation levels. In Philadelphia, we modeled declining student participation over the 12 program years, from 30 to 16%, while holding population coverage constant at 55%. For Chicago, we modeled an increase in student participation over the 4 program years, from 13 to 40%, while holding coverage constant at 12.5%.

In the second set of analyses, we used the calibrated models for all three settings to explore a set of hypothetical scenarios with varying coverage levels and participation trends, and each program scenario implemented over a 12-year period. Population coverage of school-based screening among ages 15–18 years was varied from 20 to 60% across cities. Three different time trends for participation were modeled: 1) stable participation at 50%; 2) declining participation, from 50 to 8%; and 3) increasing participation, from 50 to 90%.

In both sets of analyses, parameter uncertainty was captured by running multiple model simulations, each based on sampling one set of parameter values from the joint posterior distribution of the parameters estimated through calibration. For each draw of model parameter values, we ran both a baseline (no-screening) scenario and each of the different program scenarios. Intervention effects are summarized in terms of prevalence in different scenarios among 15–18 year-olds as well as absolute differences in prevalence between intervention scenarios and the no-screening program baseline. Reduction in prevalence was calculated by taking the difference from each baseline draw and its respective counterfactual, and calculating an overall reduction across the reductions at the draw-level. We report the median and 95% credible interval for each outcome.

## Results

Model calibration to the three settings produced baseline prevalence estimates in the absence of school-based screening. Figures S1, S2, S3 (Supplement 1) present calibration results for each setting. In 2002, the baseline prevalence estimates from the model for sexually active males and females aged 15–18 years were 5.07% (95% credible interval: 4.42–5.51%) in Philadelphia, 7.08% (3.09–9.45%) in Chicago, and 1.92% (1.52–2.44%) in rural Iowa.

### Model simulations based on features from existing school-based screening programs

**Fig. 2:**
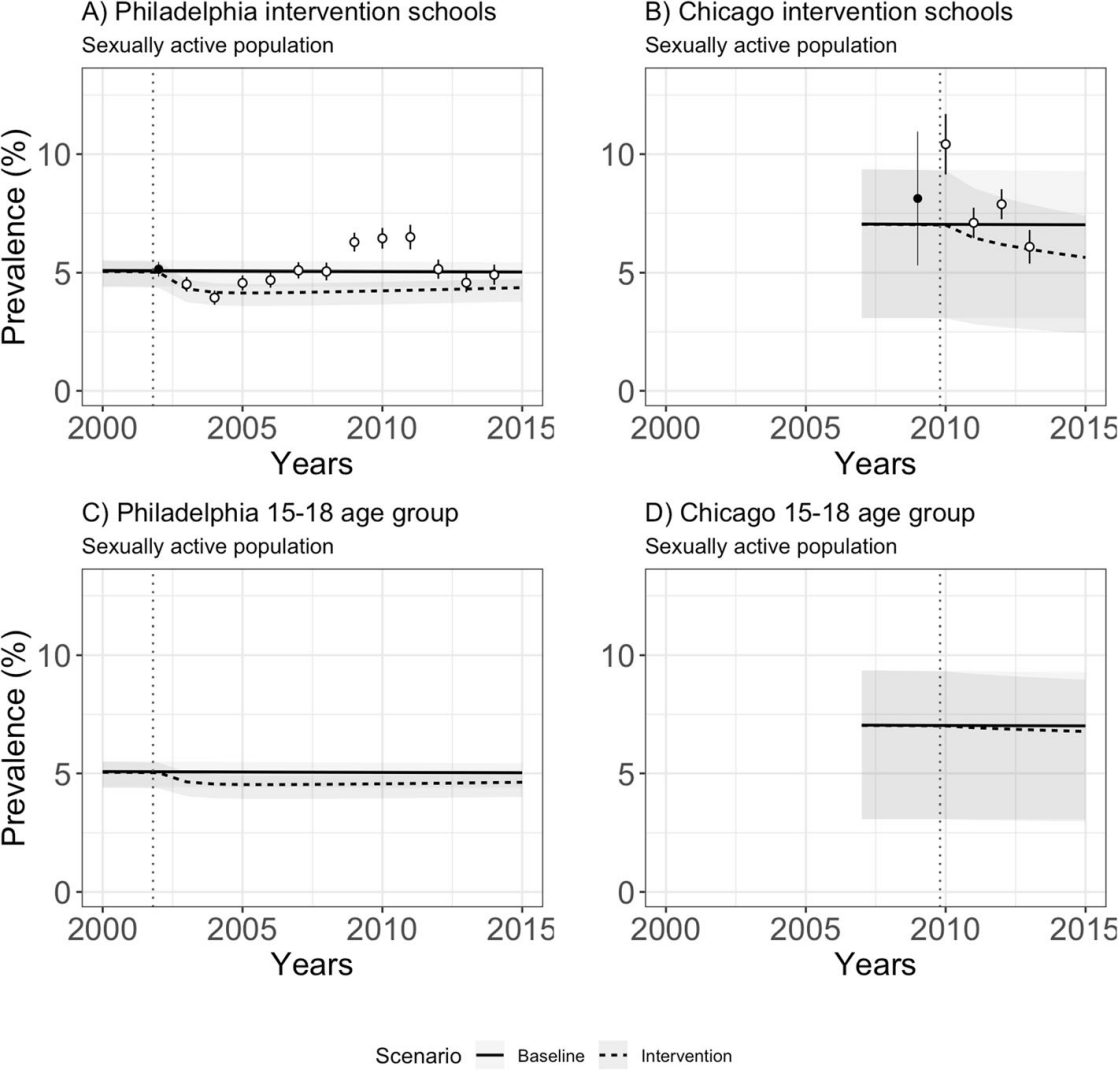
Model-estimated prevalence among sexually active students in intervention schools and in the 15–18 year-old population in the broader communities: baseline (no-screening) and intervention scenarios. Footnote: The model was calibrated to the first positivity estimate available from each program (black dot) with the positivity estimates from later years (white dots) shown for comparison. The intervention start year is marked with a dotted vertical line

Our first set of analyses simulated observed programmatic features from the school-based screening programs in high-burden cities with existing programs (Philadelphia and Chicago). Figure 2 shows the estimated prevalence for the sexually active 15–18 year-old population within intervention schools and within the surrounding communities. In 2014, after 12 years of program implementation, the model for Philadelphia predicted a prevalence of 4.34% (3.75–4.71%) among sexually active 15–18 year-old students in the intervention schools and 5.03% (4.39–5.43%) in the absence of the program, representing a 0.65 percentage point (0.57–0.71) absolute reduction. A reduction in prevalence was predicted in the initial period of the program when the student participation was at its highest, followed by a slight increase in prevalence as student participation declined (from 30 to 16%). For instance, after 4 years, the estimated prevalence in schools was 4.14% (3.59–4.53%), corresponding to a 0.88 percentage point (0.77–0.94) decrease in chlamydia prevalence. In Chicago in 2013 ─ 4 years after the program was launched ─ the prevalence in the sexually active population was estimated as 5.97% (2.60–7.88%) in the intervention schools and 7.00% (3.08–9.29%) in the absence of the program, corresponding to a 1.03 percentage point (0.48–1.40) reduction in prevalence.

At the end of the available data period for each program, the model estimated a modest population-level impact for the sexually active 15–18 year-old population in each city. In Philadelphia in 2014, chlamydia prevalence in this group was 4.61% (3.99–5.00%) compared to 5.03% (4.39–5.42%) in the absence of the program, representing a 0.38 percentage point (0.33–0.43) reduction. In Chicago in 2013, chlamydia prevalence was estimated as 6.85% (3.02–9.08%) compared to 7.00% (3.08–9.29%) in the absence of the program, representing a modest 0.16 (0.06–0.21) percentage point reduction.

### Model simulations of school-based screening in hypothetical scenarios

Our second set of analyses used models for all three settings to explore an array of hypothetical scenarios implemented over a 12-year period. Table 1 presents model-estimated prevalence for the 15–18 year-old population within intervention schools and in the broader community. Results in each case are shown separately for the full population and for the subset of sexually active students.

**Table 1:** Model-predicted prevalence among 15–18 years olds in intervention schools and in broader community over a 12-year period: baseline and intervention scenarios at the end^a^

	In intervention schools		In broader community	
	All students	Sexually active students	All people	Sexually active people
***Philadelphia***				
Prevalence in baseline scenario (%)	3.10 (2.78–3.44)	5.03 (4.39–5.42)	3.10 (2.78–3.44)	5.03 (4.39–5.42)
Reduction in prevalence with screening^b^				
At 30% coverage, with stable participation	0.85 (0.74–0.95)	1.38 (1.22–1.49)	0.28 (0.24–0.32)	0.45 (0.39–0.50)
At 30% coverage, with declining participation	0.15 (0.12–0.17)	0.24 (0.20–0.27)	0.05 (0.04–0.07)	0.08 (0.07–0.10)
At 30% coverage, with increasing participation	1.28 (1.12–1.42)	2.09 (1.84–2.23)	0.42 (0.36–0.48)	0.68 (0.59–0.75)
At 20% coverage, with stable participation	0.84 (0.73–0.94)	1.37 (1.21–1.48)	0.19 (0.16–0.21)	0.30 (0.26–0.34)
At 40% coverage, with stable participation	0.86 (0.75–0.96)	1.40 (1.23–1.50)	0.37 (0.32–0.43)	0.60 (0.52–0.67)
At 60% coverage, with stable participation	0.87 (0.76–0.98)	1.42 (1.25–1.53)	0.55 (0.48–0.63)	0.89 (0.78–0.99)
***Chicago***				
Prevalence in baseline scenario (%)	4.12 (2.07–5.48)	7.01 (3.09–9.28)	4.12 (2.07–5.48)	7.01 (3.09–9.28)
Reduction in prevalence with screening^b^				
At 30% coverage, with stable participation	1.26 (0.62–1.76)	2.15 (0.93–2.85)	0.53 (0.22–0.82)	0.91 (0.33–1.31)
At 30% coverage, with declining participation	0.26 (0.12–0.42)	0.47 (0.19–0.68)	0.14 (0.05–0.26)	0.23 (0.08–0.42)
At 30% coverage, with increasing participation	1.85 (0.92–2.54)	3.17 (1.36–4.17)	0.77 (0.32–1.16)	1.31 (0.48–1.87)
At 20% coverage, with stable participation	1.21 (0.61–1.65)	2.05 (0.91–2.73)	0.36 (0.15–0.55)	0.61 (0.22–0.89)
At 40% coverage, with stable participation	1.31 (0.63–1.86)	2.24 (0.94–3.02)	0.70 (0.29–1.08)	1.20 (0.44–1.73)
At 60% coverage, with stable participation	1.44 (0.65–2.07)	2.42 (0.97–3.39)	1.04 (0.43–1.58)	1.76 (0.64–2.54)
***Rural Iowa***^*c*^				
Prevalence in baseline scenario (%)	0.58 (0.41–0.7)	1.28 (0.92–1.54)	0.58 (0.92–0.72)	1.28 (0.92–1.54)
Reduction in prevalence with screening^b^				
At 30% coverage, with stable participation	0.18 (0.13–0.22)	0.40 (0.29–0.47)	0.07 (0.05–0.09)	0.16 (0.12–0.19)
At 30% coverage, with declining participation	0.04 (0.03–0.05)	0.09 (0.07–0.11)	0.02 (0.02–0.03)	0.05 (0.04–0.06)
At 30% coverage, with increasing participation	0.26 (0.18–0.32)	0.58 (0.42–0.69)	0.10 (0.08–0.12)	0.23 (0.17–0.27)
At 20% coverage, with stable participation	0.17 (0.12–0.21)	0.38 (0.28–0.46)	0.05 (0.04–0.06)	0.11 (0.08–0.13)
At 40% coverage, with stable participation	0.19 (0.13–0.23)	0.41 (0.30–0.48)	0.10 (0.07–0.11)	0.21 (0.15–0.25)
At 60% coverage, with stable participation	0.20 (0.14–0.24)	0.44 (0.32–0.51)	0.14 (0.10–0.17)	0.31 (0.23–0.36)

^a^All results show median values and 95% credible intervals

^b^Reductions are expressed in absolute terms, i.e. as percentage point decreases in prevalence

^c^Rural Iowa was simulated using the state-level diagnosis reports and excluding the 10 most populous counties

For a school-based screening program covering 30% of the age group, and which maintained stable student participation, we estimated that the program would reduce prevalence among sexually active students in the intervention schools by 1.38 (1.22–1.49), 2.15 (0.93–2.85) and 0.40 (0.29–0.47) percentage points in Philadelphia, Chicago and Iowa, respectively. If student participation declined over time, benefits compared to baseline would shrink: reduction of 0.24 (0.20–0.27) percentage points in Philadelphia, 0.47 (0.19–0.68) percentage points in Chicago, and 0.09 (0.07–0.11) percentage points in Iowa. With increasing student participation, the reductions in prevalence in the three settings would be 2.09 (1.84–2.23), 3.17 (1.36–4.17) and 0.58 (0.42–0.69) percentage points, respectively.

Holding participation constant but varying coverage from 30 to 60% of the 15–18 year-old population demonstrates the potential impact of doubling the size and reach of the program. Given the same level of participation within intervention schools, program effects expressed as reductions in prevalence among the students in intervention schools were relatively invariant to the overall population coverage of the program. On the other hand, considering the effects in the broader communities around the participating schools, with a 30% program coverage there was an estimated 0.45 (0.39–0.50) percentage point decrease in community prevalence among the sexually active population in Philadelphia, a 0.91 (0.33–1.31) percentage point decrease in Chicago, and a 0.16 (0.12–0.19) percentage point decrease in rural Iowa. With a 60% program coverage the corresponding decreases were 0.89 (0.78–0.99), 1.76 (0.64–2.54) and 0.31 (0.23–0.36) percentage points, respectively.

## Discussion

In this study, we examined the impact of existing and hypothetical school-based chlamydia screening interventions on the prevalence in intervention schools and surrounding communities. Our results demonstrated that stable and sustained school-based screening programs are needed to maximize and maintain the impact of the interventions. In the Philadelphia intervention implemented during 2002–2014, we predicted an initial decline in chlamydia positivity. Although these initial declines were not entirely sustained, we estimated an overall reduction in prevalence compared to no school-based program baseline scenario after 12 years of program implementation. Reducing student participation in our model to the levels observed in some existing programs led to modelled prevalence estimates consistent with observed trends. Program coverage, measured in terms of the proportion of the 15–18 years old population enrolled in participating schools, was larger in Philadelphia than in Chicago, which resulted in larger population-level benefits in Philadelphia. In all hypothetical scenarios, school-based chlamydia screening had a larger impact in higher burden settings (modeled after Chicago and Philadelphia) than in a lower burden setting (modeled after rural Iowa).

Schools have been a venue for the delivery of complex sexual and reproductive health interventions such as sex education initiatives [26] and risk behavior reduction programs [27]. School-based STI screening initiatives can offer opportunities for diagnosis and treatment of STIs among adolescents who may not be able to access sexual health services elsewhere, and therefore reduce time to treatment [14]. A previous study which examined screening in New Orleans high schools during the 1997–1998 school year indicated that the program could be cost-effective and cost-saving [28].

Sustaining school-based screening requires considerable resources at the local level [9, 14]. All screening efforts require funding, infrastructure, and human resources. Using Chicago and Philadelphia as examples: Philadelphia has established a stable, wide-spread program, and Chicago had a successful pilot and roll-out of school-based screening. In 2015, the Chicago Department of Public Health partnered with Planned Parenthood Illinois, which now provides sexual health education and STI screening in the school-based screening program. This partnership increased program capacity and facilitated the inclusion of broader services. Both cities have opt-out consent instead of participation requiring active parental consent. In Philadelphia, condoms are available, partner notification is provided, and treatment is provided in schools for people testing positive for chlamydia. In Chicago, condoms are made available at the discretion of the principal of each school, and access varies across the school district. It is likely that there are further benefits from these programs to individuals and to the communities than those captured in this study. There are a number of programs in the country, which operate in a similar fashion: for example, Michigan’s program in Detroit has sustained its screening program through a partnership between the state health department, schools, and the local health system. In Detroit, students are offered partner notification, and partners of students can receive services in any school-based health center, which also function as community health centers.

School-based screening programs often have limited information available to evaluate the impact of the intervention in the surrounding communities. As this mathematical modeling study demonstrated, school-based programs can theoretically reduce broader community STD rates. However, positivity trends are not directly comparable without understanding how the program was implemented and what coverage level was attained [8, 9]. Different program coverage levels can result in a similar impact within the schools taking part in the program, but a different impact for the community as indicated in the results by varying the intervention coverage in the age-population. To further understand how the programs operate, data on how many students participated in more than one screening event and on potential differences between students who take up screening and those who do not would be useful. As a limitation of the study, we did not have access to data on how many students participated in more than one screening, nor did our model-structure allow for analysis of repeat screening of a subset of the intervention school population. Previous work [6] has suggested that repeat yearly student participation in screening is difficult to sustain. There are also features of sexual networks, such as where the students find partners [11], that can further influence the success of the programs. Features of sexual networks are likely highly dependent on community-level factors, such as interconnectedness of city neighborhoods and availability of public transportation, which may facilitate different mixing patterns among the young people compared to the patterns in cities that are less connected [11, 16]. For rural communities, there is limited information on mixing patterns among adolescents.

Our model calibration was limited by the availability of data at the local level. We relied mostly on reported chlamydia diagnoses as a measure of chlamydia burden. We assumed that screening at schools would not influence the students’ screening outside of the program (i.e., that the interventions were additive to existing services). If screening at schools made it more likely that students would not get screened in the community, this would lower the overall impact of the intervention compared to the results in our study. Conversely, if students were better informed due to the availability of school-based screening, they may be more likely to use community services in the future, which would increase the impact of the intervention. Once exposed to the opportunity to undergo STI screening, young people may have an increased awareness of their ability or right to access confidential health services, and they may seek out other sexual health services, including expanded HIV/STI testing or reproductive services, as has been suggested before [14]. Overall, there is a lack of evidence on how school-based screening interventions impact students’ current and future engagement with sexual health services, and this question remains an important priority for future research.

The highest burden of STIs remains in urban areas, where young people are a key population for STI control strategies. Interventions that can reach people outside of traditional clinic settings are an important component of comprehensive sexual health programs. Young people may not use sexual health services due to a lack of information regarding their rights to access these services on their own, concerns regarding privacy, cost, transportation, or some combination of these [4]. By offering access to health care interventions within the school setting, programs such as school-based STI screening can mitigate utilization constraints related to education, transportation, and cost.

## Conclusions

To maximize the school-based screening programs’ impact on students and communities at large, a stable program with high participation among high school students is likely needed. Targeting the population with the highest burden of chlamydia will allow for the identification of the most infections, and will have a greater influence on chlamydia transmission dynamics.

## Supplementary information

**Additional file 1: Table S1.** Parameter table describing the input parameters, and posterior parameter estimates. **Figure S1.** Calibration of the model to Philadelphia. **Figure S2.** Calibration of the model to Chicago. **Figure S3.** Calibration of the model to Rural-Iowa.

## Data Availability

Each local public health insititution decides on their data-sharing policy, and for any data related to school-based screening, they should be contacted for data access. City level chlamydia surveillance data is publicly available. Model code is available on request from the corresponding author.
