# Supplementary material for: Mathematical modeling study of school-based chlamydia screening: potential impact on chlamydia prevalence in intervention schools and surrounding communities
Source: BMC Public Health. 2020 Sep 5;20:1363. doi: 10.1186/s12889-020-09466-y (PMC7487537; doi:10.1186/s12889-020-09466-y)
Supplement: Supplementary file 1 — Additional file 1: Table S1. Parameter table describing the input parameters, and posterior parameter estimates. Figure S1. Calibration of the model to Philadelphia. Figure S2. Calibration of the model to Chicago. Figure S3. Calibration of the model to Rural-Iowa. [file 12889_2020_9466_MOESM1_ESM.pdf]

Supplementary material

The potential impact of chlamydia screening in high schools on chlamydia transmission dynamics

Table S1. Parameter table describing the input parameters, and posterior parameter estimates

		Prior distribution	Posterior distribution (median and 95% range)			
		Input parameter	**Philadelphia**	**Chicago**	**Rural-Iowa**	Source
**Parameter/ Variable**	**Description**					
**Population size**	Setting-specific	Fixed	1.6 million	2.7 million	1.5 million **	(1)
**Time step**	Timestep used in the model simulations	Fixed	1 wk	1 wk	1 wk	
**Testing symptomatic individuals (pw)**						
Women	Testing of symptomatic women	0.183 (0.179-0.192)	0.158 (0.154-0.168)	0.157 (0.145-0.193)	0.191(0.186-0.198)	Resampled (2,3)
Men	Testing of symptomatic men	0.171 (0.165-0.177)	0.169 (0.156-0.178)	0.158 (0.14-0.186)	0.153 (0.15-0.16)	Resampled (2,3)
**Testing asymptomatic 15-24 years old women (pw)**						
	Start(2000)	Beta(1,1200)	0.001 (0-0.001)	0.001 (0-0.001)	0 (0-0)	(2)
	End (2015)	Beta(15,1000)	0.001 (0-0.001)	0.001 (0-0.001)	0.006 (0.006-0.007)	‘’
**Testing asymptomatic 25-39 women (pw)**						
	Start (2000)	Beta(1,1400)	0 (0-0.001)	0 (0-0.001)	0 (0-0)	(2)
	End (2015)	Beta(4,1200)	0 (0-0.001)	0 (0-0.001)	0.002 (0.001-0.002)	‘’

**Screening in men**						
Men 15-18, 19-24	Scaler vs the same age group of women	Beta(10,70)	0.062 (0.048-0.079)	0.122 (0.04-0.217)	0.054 (0.04-0.059)	(2)
Men 25-39	Scaler vs W, aged 25-39	Beta(10,100)	0.027 (0.018-0.036)	0.035 (0.009-0.05)	0.04 (0.038-0.051)	‘’
**Partner notification**						
Women						
	PN, index case F 19-24	0.689 (0.633-0.777)	0.697 (0.561-0.757)	0.679 (0.63-0.769)	0.682 (0.628-0.759)	Resampled (2)
	PN, index case F 25-39	0.534 (0.488-0.58)	0.557 (0.499-0.586)	0.535 (0.487-0.591)	0.539 (0.51-0.57)	‘’
	PN, index case F 40-54	0.505 (0.472-0.54)	0.51 (0.468-0.561)	0.502 (0.484-0.525)	0.502 (0.473-0.545)	‘’
Men						
	PN, index case M 19-24	0.656 (0.572-0.704)	0.722 (0.652-0.771)	0.659 (0.522-0.7)	0.643 (0.579-0.698)	Resampled (2)
	PN, index case M 25-39	0.766 (0.72-0.802)	0.749 (0.708-0.835)	0.764 (0.738-0.793)	0.778 (0.742-0.802)	‘’
	PN, index case M 40-54	0.719 (0.665-0.765)	0.721 (0.678-0.842)	0.723 (0.663-0.761)	0.707 (0.675-0.748)	‘’
**Test sensitivity**						
	Sensitivity of NAAT	0.964 (0.937-0.982)	0.95 (0.934-0.981)	0.965 (0.938-0.981)	0.964 (0.938-0.984)	Resampled (2)
	Sensitivity of non NAAT	0.999 (0.993-1)	0.997 (0.993-1)	0.998 (0.993-1)	0.999 (0.995-1)	‘’
**Treatment success**						
	Index case	0.954 (0.947-0.959)	0.954 (0.95-0.961)	0.954 (0.948-0.958)	0.955 (0.948-0.959)	Resampled (2)
	Partner of index case	0.959 (0.955-0.963)	0.96 (0.957-0.963)	0.959 (0.956-0.963)	0.959 (0.955-0.962)	‘’
**Reporting**	Proportion of diagnosed cases reported per year.					
Women	Proportion of diagnosed cases reported in 2000	Beta(7,3)	0.919 (0.89-0.94)	0.826 (0.717-0.926)	0.606 (0.531-0.623)	Assumption
Men	Relative to women	Beta(8,2)	0.174 (0.153-0.19)	0.618 (0.526-0.718)	0.701 (0.681-0.79)	‘’
**Natural recovery (pw)**						
Women		0.015 (0.015-0.016)	0.015 (0.015-0.015)	0.015 (0.015-0.016)	0.015 (0.015-0.016)	Resampled (2–4)
Men		0.016 (0.015-0.016)	0.016 (0.016-0.016)	0.016 (0.015-0.016)	0.016 (0.016-0.016)	‘’
**Transmission probability**						
	Per act probability	0.021 (0.019-0.026)	0.018 (0.017-0.02)	0.022 (0.02-0.026)	0.021 (0.02-0.023)	Resampled (2,4)
	Relative	Fixed	2	2	2	‘’

	transmission probability increase from men to women					
**Proportion with symptoms**						
Women	Proportion symptomatic	0.287 (0.281-0.297)	0.291 (0.284-0.295)	0.288 (0.282-0.295)	0.29 (0.284-0.296)	Resampled (2,3)
Men		0.262 (0.252-0.273)	0.259 (0.234-0.264)	0.262 (0.251-0.27)	0.263 (0.253-0.273)	‘’
**Sexual debut**						
Women	Had sex before age 15	Beta(5,5.55)	0.349 (0.275-0.413)	0.347 (0.224-0.463)	0.267 (0.047-0.345)	(5–7)
	Rate of sexual initiation, calculated from:					
	proportion sexually active by 18 (of those not yet active) used to derived a rate	Beta(8,11)	0.595 (0.536-0.647)	0.428 (0.321-0.576)	0.427 (0.374-0.44)	‘’
	Relative change in sexual debut	Uniform(0.77-1.3)	0.796 (0.774-0.858)	0.891 (0.781-1.166)	0.956 (0.873-1.004)	‘’
	Rate of sexual initiation, calculated from the					
	proportion sexually active by 25 (of those not yet active) used to derived a rate	0.93 (0.922-0.942)	0.934 (0.925-0.938)	0.92 (0.911-0.938)	0.92 (0.908-0.926)	Resampled (2)
Men	Had sex before age 15	Beta(5,5.55)	0.37 (0.301-0.434)	0.409 (0.321-0.546)	0.071 (0.002-0.252)	(5–7)
	Rate of sexual initiation, calculated from:					
	proportion sexually active by 18 (of those not yet active) used to derived a rate	Beta(8,11)	0.548 (0.493-0.593)	0.475 (0.332-0.627)	0.425 (0.416-0.466)	‘’
	Relative change in sexual debut	Uniform(0.77-1.3)	1.065 (0.955-1.102)	1.021 (0.921-1.184)	1.061 (1.02-1.07)	‘’
	Rate of sexual initiation, calculated from:					
	proportion sexually active by 25 used to derived a rate	0.93 (0.922-0.942)	0.925 (0.916-0.943)	0.93 (0.924-0.942)	0.929 (0.924-0.939)	Resampled (2)
**Casual**						

**partners (pw)**						
High-risk (HR)	Single, 15-18 HR	Beta(3,30)	0.053 (0.037-0.066)	0.149 (0.049-0.258)	0.085 (0.081-0.124)	(7); Assumption
	Single, 19-24 HR	Beta(3,30)	0.128 (0.116-0.14)	0.1 (0.052-0.144)	0.155 (0.146-0.166)	‘’
	Single, 25-39 HR	Beta(3,60)	0.054 (0.045-0.06)	0.073 (0.029-0.107)	0.043 (0.038-0.055)	‘’
	Single, 40-54 HR	Beta(3,400)	0.002 (0.001-0.003)	0.006 (0.003-0.01)	0 (0-0.001)	‘’
Low-risk (LR)	Single, 15-18 LR	Beta(1,40)	0.01 (0.007-0.015)	0.022 (0.006-0.045)	0.008 (0.004-0.012)	‘’
	Single, 19-24 LR	Beta(1,40)	0.17 (0.158-0.177)	0.089 (0.063-0.114)	0.071 (0.069-0.075)	‘’
	Single, 25-39 LR	Beta(3,30)	0.018 (0.016-0.021)	0.007 (0.002-0.012)	0.002 (0-0.003)	‘’
	Single, 40-54 LR	Beta(3,30)	0 (0-0)	0.001 (0-0.002)	0 (0-0)	‘’
Among paired (concurrent)	Relative rate compared to the single group					
High-risk		Beta(10,70)	0.098 (0.074-0.119)	0.153 (0.101-0.205)	0.097 (0.092-0.126)	‘’
Low-risk		Beta(10,100)	0.115 (0.098-0.134)	0.074 (0.052-0.143)	0.093 (0.078-0.11)	‘’
	Age mixing for women W 19-24	Fixed	0.2	0.2	0.2	(2) Assumed the same as at national level
	Age mixing for women 25-39	Fixed	0.98	0.98	0.98	‘’
	Age mixing for men 19-24	Fixed	0.99	0.99	0.99	‘’
	Age mixing for men 25-39	Fixed	0.5	0.5	0.5	‘’
	Age mixing for men 40-54	Fixed	0.99	0.99	0.99	‘’
	Pair formation rate 19-24 F&M	Fixed	0.002	0.002	0.002	‘’
	Pair formation rate 25-39 F&M	Fixed	0.004	0.004	0.004 (	‘’
	Pair formation rate 40-55 F&M	Fixed	0.007	0.007	0.007	‘’

Footnote:
W: Women; M: Men; py: per year; pw per week; HR: high risk; LR: low risk

** The population in rural-Iowa was approximated by removing the 10 most populous counties from the population estimate.

Figure S1. Calibration of the model to Philadelphia

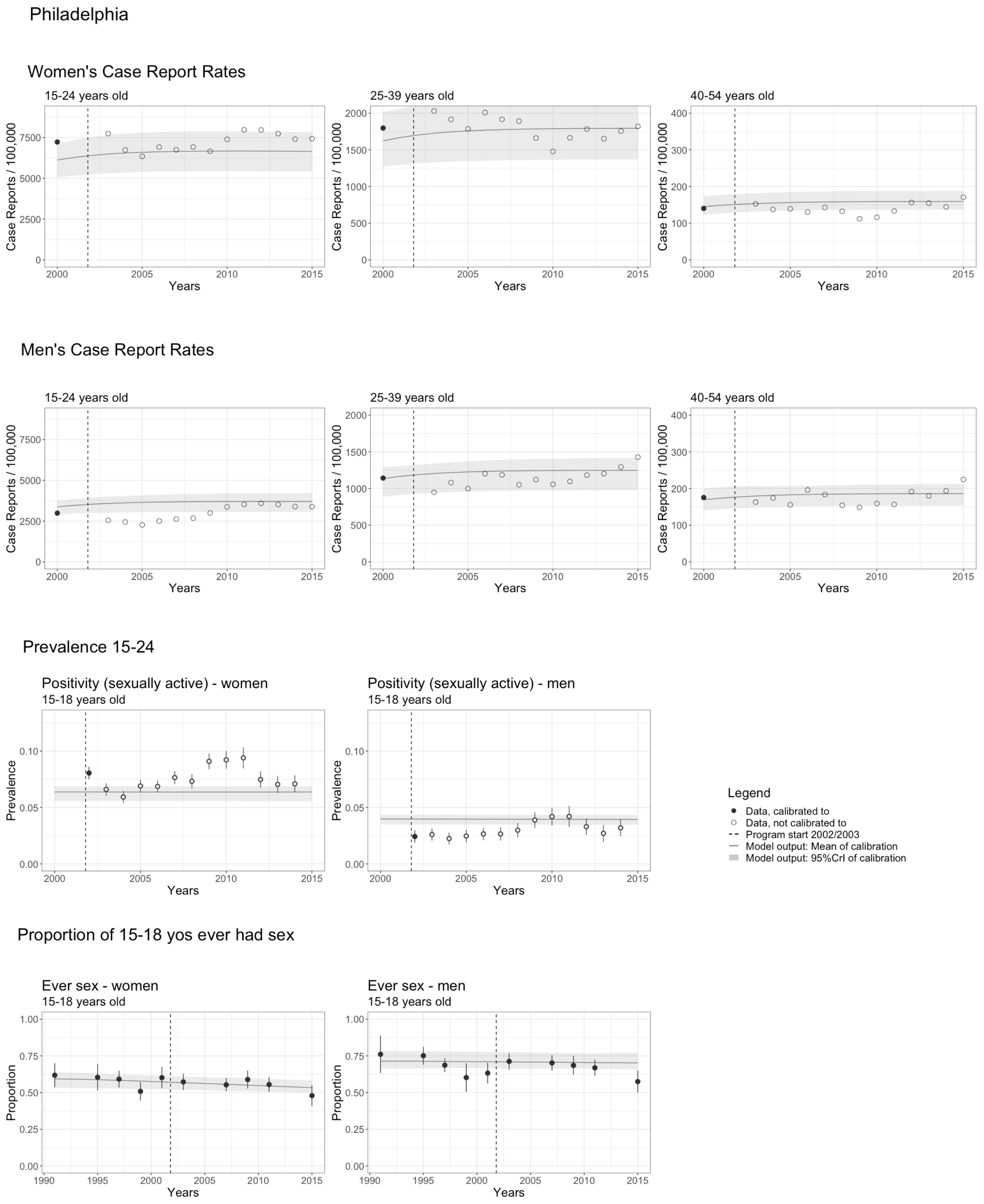

Figure S2. Calibration of the model to Chicago

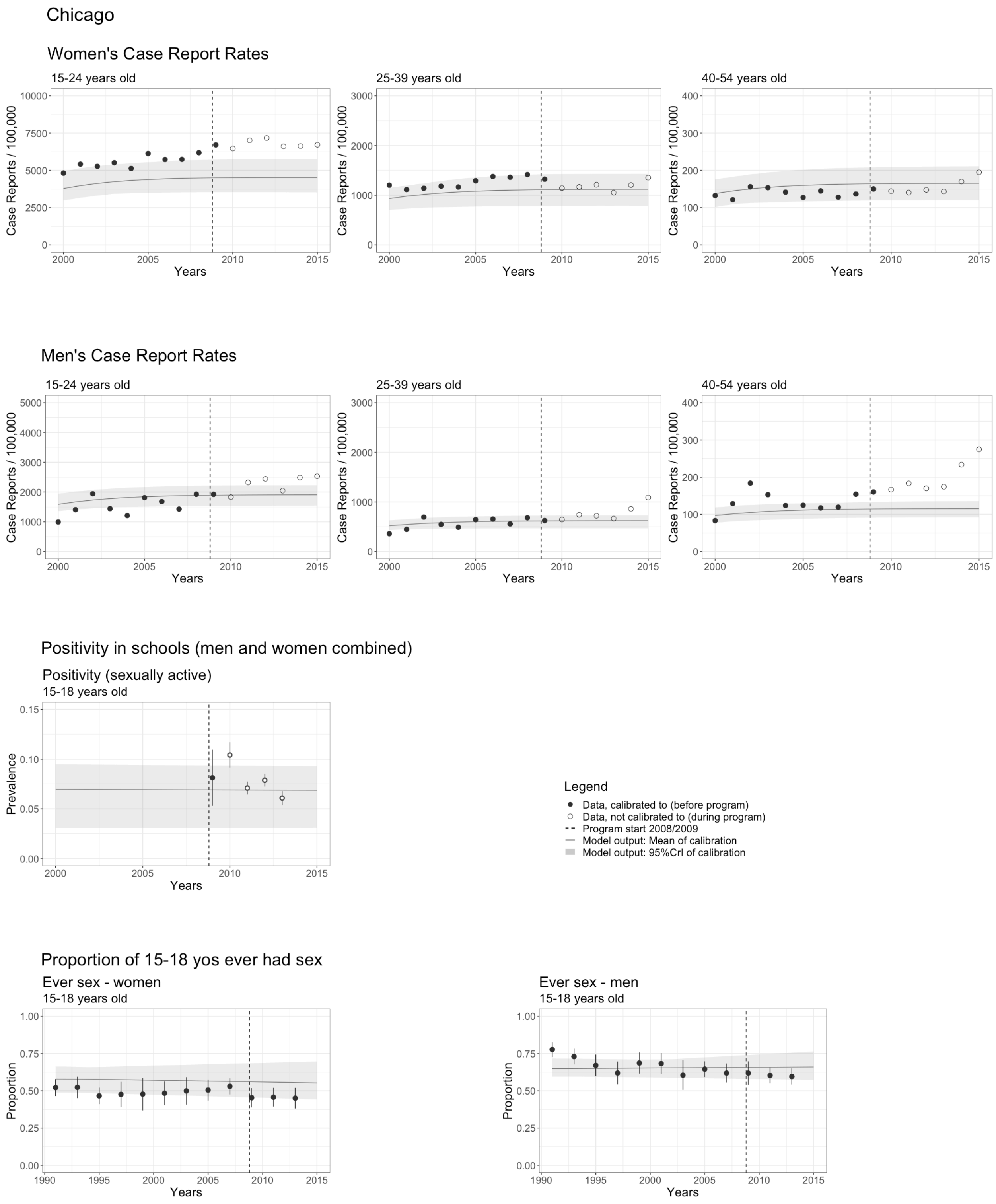

Figure S3. Calibration of the model to Rural-Iowa

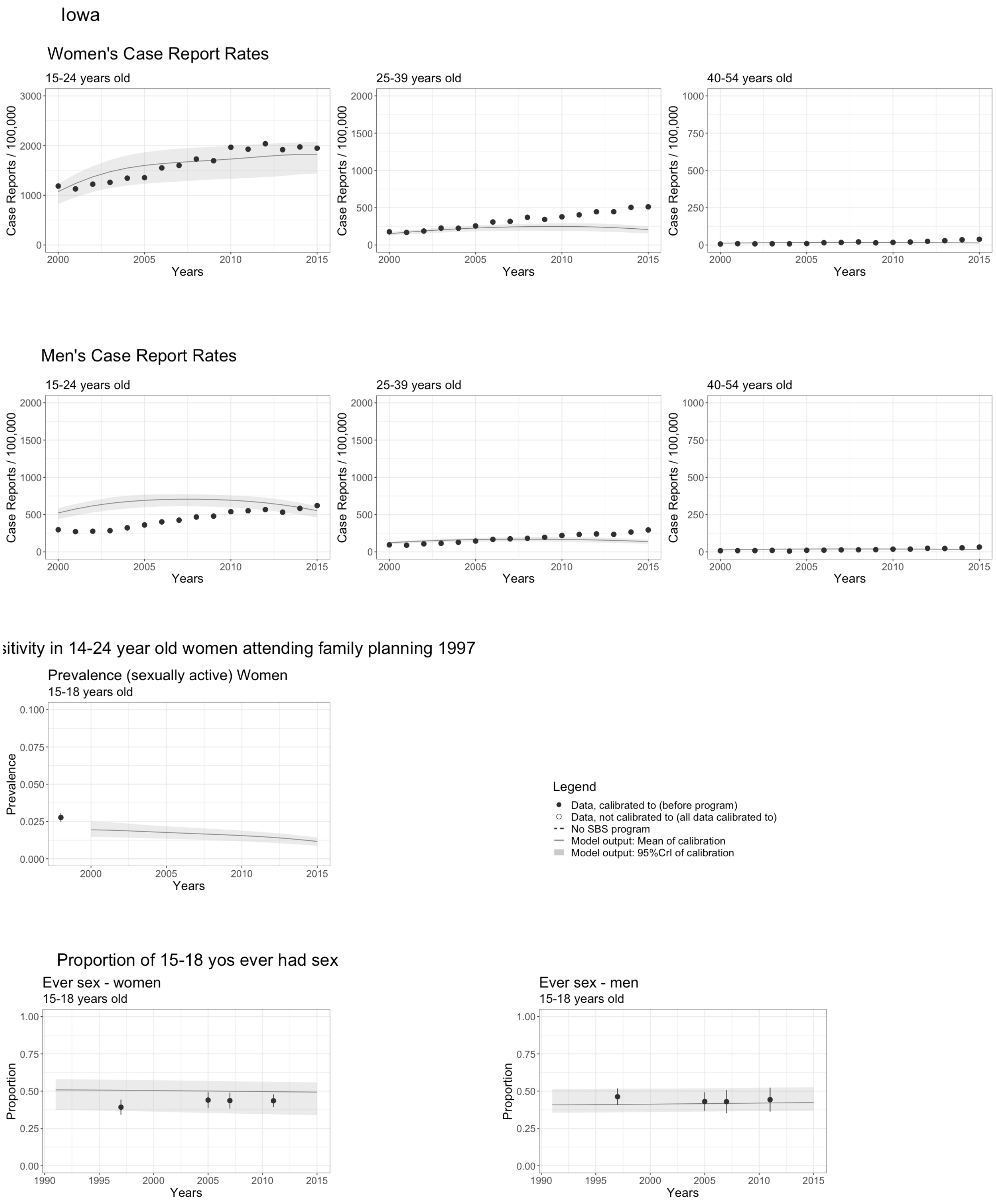

References

1. U.S. Census Bureau DIS. Age and Sex Composition in the United States: 2012.
2. Rönn MM, Tuite AR, Menzies NA, Wolf EE, Gift TL, Chesson HW, et al. The impact of screening and partner notification on chlamydia in the United States, 2000 to 2015: evaluation of epidemiological trends using a pair-formation transmission model. Am J Epidemiol. 2018;Accepted.
3. Price MJ, Ades AE, Soldan K, Welton NJ, Macleod J, Simms I, et al. The natural history of Chlamydia trachomatis infection in women: a multi-parameter evidence synthesis. Health Technol Assess. NIHR Journals Library; 2016 Mar;20(22):1–250.
4. Davies B, Anderson S-J, Turner KME, Ward H. How robust are the natural history parameters used in chlamydia transmission dynamic models? A systematic review. Theor Biol Med Model. Theoretical Biology and Medical Modelling; 2014 Jan 30;11(1):8.
5. Centers for Disease Control and Prevention. Youth Risk Behavior Survey Questionnaire.
6. Centers for Disease Control and Prevention (CDC). National Center for Health Statistics (NCHS). National Health and Nutrition and Examination Survey Data. [Internet]. Hyattsville, MD: U.S. Department of Health and Human Services, Centers for Disease Control and Prevention; Available from: https://wwwn.cdc.gov/nchs/nhanes/default.aspx
7. Centers for Disease Control and Prevention (CDC). National Center for Health Statistics (NCHS). National Survey of Family Growth. U.S. Department of Health and Human Services, Centers for Disease Control and Prevention;
